# Effect of Zn Content on the Microstructure and Mechanical Properties of Mg–Al–Sn–Mn Alloys

**DOI:** 10.3390/ma12193102

**Published:** 2019-09-23

**Authors:** Tianshuo Zhao, Yaobo Hu, Fusheng Pan, Bing He, Maosheng Guan, Yuan Yuan, Aitao Tang

**Affiliations:** 1 College of Materials Science and Engineering, Chongqing University, Chongqing 400044, China; 2 National Engineering Research Center for Magnesium Alloys, Chongqing 400044, China

**Keywords:** magnesium alloy, Zn content, extrusion, tensile property

## Abstract

High performance Mg–6Al–3Sn–0.25Mn–*x*Zn alloys (*x* = 0, 0.5, 1.0, 1.5, and 2.0 wt %) without rare earth were designed. The effects of different Zn contents on the microstructure and mechanical properties were systematically investigated. The addition of Zn obviously refines the as-cast alloys dendritic structure because of the increase in the number in the second phase. For the as-extruded alloys, an appropriate amount of Zn promotes complete recrystallization, thus increasing the grain size. As the Zn content increases, the texture gradually evolves into a typical strong basal texture, which means that the basal slip is difficult to initiate. Meanwhile, the addition of Zn promotes the precipitation of small-sized second phases, which can hinder the dislocation movement. The combination of texture strengthening and precipitation strengthening is the main reason for the improvement of alloys’ strength.

## 1. Introduction

Magnesium alloys are considered as promising structural materials in the automotive and aerospace industry because of their low density, high stiffness, and easy recycling [1,2,3]. But, at present, magnesium alloys are not widely used and cannot completely replace steel and aluminum alloys. This is because the specific strength of the magnesium alloy is very high, but the absolute strength is relatively low, which makes it difficult to apply to large-scale engineering fields [4]. Therefore, the improvement of the alloy strength is one of the most important research directions. In general, the introduction of rare earth (RE) elements into Mg alloys can significantly improve the mechanical properties of an alloy [5,6,7]. However, RE elements are expensive, which is not conducive to the wide application of magnesium alloys. It is therefore necessary to develop a series of rare earth-free and high-performance magnesium alloys.

Mg–Al-based alloys are the most commonly used magnesium alloy systems, with good strength, ductility, and corrosion resistance [8]. Part of the aluminum element is dissolved in the α-Mg matrix so as to achieve solid solution strengthening. Furthermore, the Mg_17_Al_12_ phase precipitated along the grain boundary enhances the strength of the magnesium alloy by precipitation strengthening. However, too much aluminum will lead to a poor ductility and other alloy elements need to be added to obtain better comprehensive mechanical properties. It has been reported that adding Sn to Mg–Al–based alloys can significantly improve their mechanical properties [9,10]. Park et al. [11] reported that the Mg–8Sn–1Al–1Zn alloy exhibits tensile and compressive strengthening compared with the commercial AZ31 alloy, which is attributed to grain refinement and the presence of the Mg_2_Sn phase. Wang et al. [12] used the first principle to calculate that the addition of Sn reduces the stacking fault energies of pyramidal slip systems. The increase of non-basal slip activity is beneficial for the improvement of elongation. Suh et al. [13] also investigated that the Mg–3Al–1Sn alloy possesses a much higher stretch formability because of the prismatic <a> slip as the dominant deformation mode. The addition of Sn promotes the formation of the thermally stable Mg_2_Sn second phase, and disperses it at the matrix and grain boundary, thereby improving the creep resistance of the magnesium alloys. Meanwhile, the precipitated Mg_2_Sn phase is advantageous for improving the extrudability of the alloys. Manganese is considered to be an effective impurity-eliminating element, and can improve the metallurgical quality of the alloys.

In addition, zinc is widely regarded as an alloy element that can effectively enhance the strength of magnesium alloys [14,15,16]. The addition of zinc can reduce the solid solubility of aluminum in the α-Mg matrix, thereby promoting the precipitation of the Mg_17_Al_12_ phase, and finally achieve the second phase strengthening. In Mg–Al-based alloys system, the ternary MgAlZn phase, will be formed, and these second phase will increase the strength of the alloy through precipitation strengthening. However, the effect of different Zn contents on Mg–Al–Sn–Mn alloy systems has not been studied systematically. In the present work, Mg–6Al–3Sn–0.25Mn–*x*Zn (*x* = 0, 0.5, 1.0, 1.5, and 2.0 wt %) alloys were prepared. The effects of Zn on the microstructure, textures, and mechanical properties of the as-extruded alloys were investigated.

## 2. Materials and Methods 

The raw materials for the experimental alloys include commercially pure magnesium (99.9 wt %), pure aluminum (99.9 wt %), pure zinc (99.9 wt %), pure tin (99.9 wt %), and MnCl_2_ (99.7 wt %). The as-cast alloys were prepared by smelting in an electromagnetic induction furnace under the protection mixture gas (CO_2_ + SF_6_) at 720 °C. The actual chemical compositions of the as-cast alloys were determined by X-ray fluorescence spectrometry (XRF-1800, Shimadzu, Kyoto, Japan), as shown in Table 1. The cast ingots (ϕ 80 mm × 45 mm) were solution heat treated at 300 °C for 12 h, followed by 12 h at 390 °C. Then, the ingots were directly extruded at 350 °C, with an extrusion ratio of 25. 

**Figure 1:**
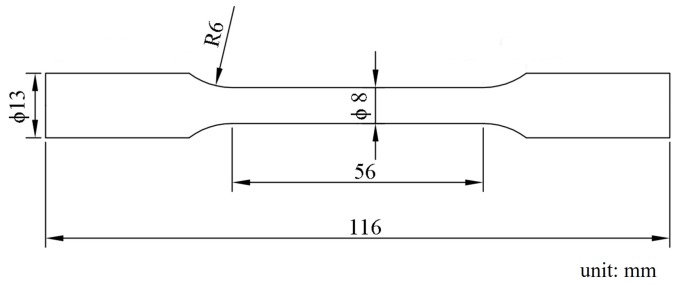
Size of the room temperature tensile samples.

The samples for the microstructure observation were ground on SiC papers and etched with an acetic-picral solution (2 g of picric acid, 3 mL of acetic acid, and 18 mL of anhydrous ethanol). The microstructures were analyzed by optical microscopy (OM, Carl Zeiss, Jena, German) and scanning electron microscope (SEM, VEGA Ⅱ LMU, TESCAN, Brno, Czech Republic). X-ray diffraction (XRD) measurements were conducted using the D/MAX-2500PC (Rigaku, Tokyo, Japan) with Cu Kα radiation. Electron backscatter diffraction (EBSD) was performed using JSM-7800F (JEOL Ltd., Tokyo, Japan) with a step size of 0.5 μm. The specimens for EBSD were ground mechanically and had electrochemical polishing in a commercial AC_2_ solution. The obtained EBSD data were analyzed by channel 5 software (Oxford Instruments, Oxford, UK). Tensile tests were carried out at room temperature by using an GMT-5105 testing machine (Xinsansi, Shenzhen, China) with a constant strain rate of 2.0 × 10^−3^ /s. The specimens were machined from extruded bars parallel to the extruded direction (ED). According to the GB/T 16865-2013 standard, the samples were processed into a dog-bone shape by wire cutting. The size of the tested specimen is shown in Figure 1, which has a diameter of 8 mm. In order to ensure the reliability of the experimental data, three tested specimens of each alloy were processed and tested. The reported results are the averages over the three tests.

## 3. Results

### 3.1. Microstructure Characterization of As-Cast Mg–6Al–3Sn–0.25Mn–xZn Alloys

**Figure 2:**
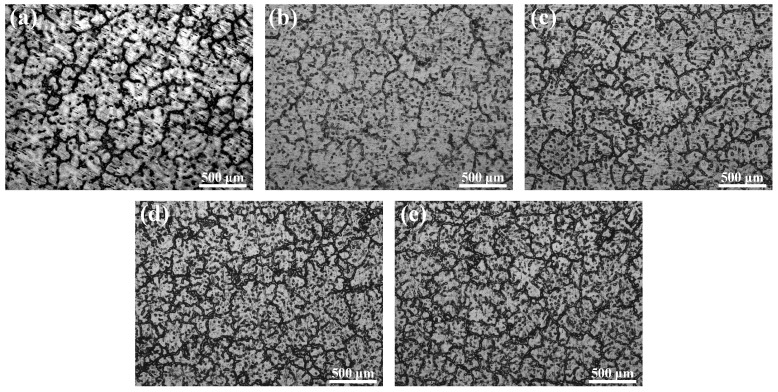
Optical microstructures of the as-cast Mg–6Al–3Sn–0.25Mn–*x*Zn alloys: (**a**) 0 wt %; (**b**) 0.5 wt %; (**c**) 1.0 wt %; (**d**) 1.5 wt %; (**e**) 2.0 wt %.

Figure 2 shows the optical micrographs of the as-cast Mg–6Al–3Sn–0.25Mn-based alloys with various Zn contents. All of the cast alloys are characteristic of a typical dendritic structure. The second phase is mainly distributed near the dendrite boundaries and inside the matrix. It is apparent that the volume fraction of the second phase increases with the addition of Zn. For Mg–6Al–3Sn–0.25Mn–*x*Zn alloys, the volume fraction of the phases is 9.6%, 11.9%, 17.7%, 22.6%, and 24.5%, respectively. It can be considered that the formed second phase effectively reduces dendrite spacing and promotes cast grain refinement [17].

**Figure 3:**
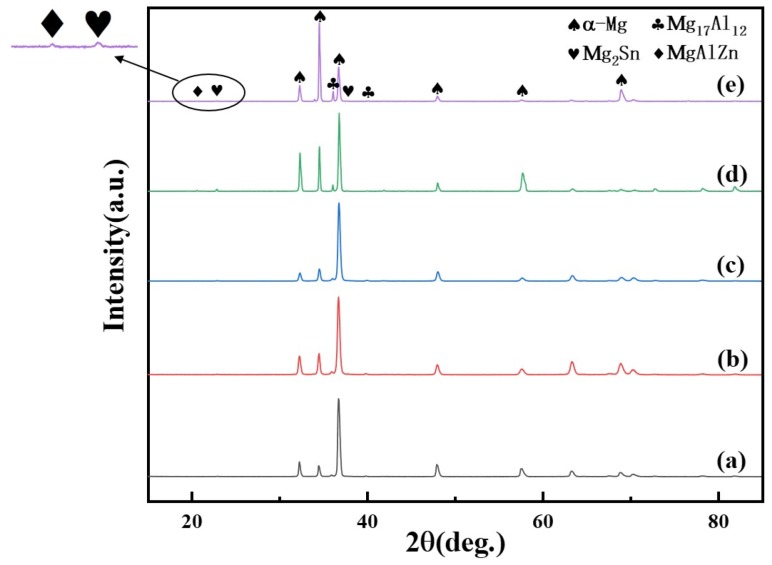
XRD patterns of the as-cast Mg–6Al–3Sn–0.25Mn–*x*Zn alloys: (**a**) 0 wt %; (**b**) 0.5 wt %; (**c**) 1.0 wt %; (**d**) 1.5 wt %; (**e**) 2.0 wt %.

**Figure 4:**
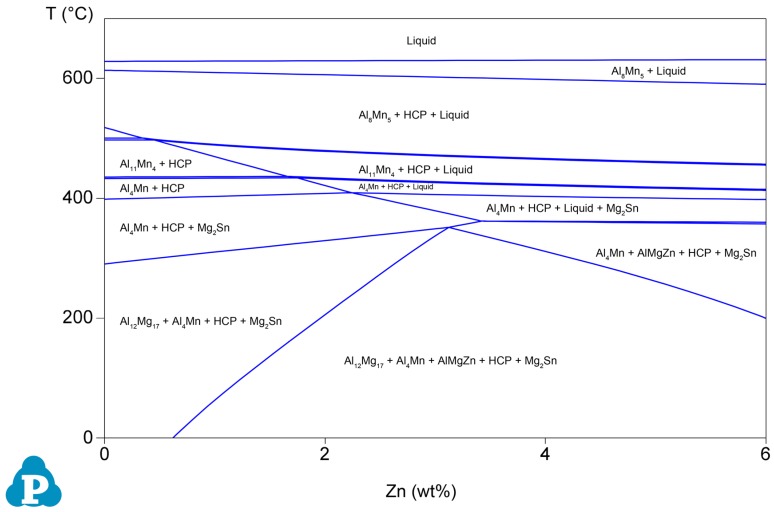
Phase diagrams of the Mg–6Al–3Sn–0.25Mn–xZn alloys.

The normalized XRD patterns of the as-cast Mg–6Al–3Sn–0.25Mn–*x*Zn alloys are presented in Figure 3. All of the as-cast alloys contain three phases: α-Mg, Mg_17_Al_12_, and Mg_2_Sn. When the addition of Zn exceeds 1.5 wt %, the diffraction peak of the MgAlZn phase begins to appear, which means that the number of the second phase containing Zn increases. Combined with the phase diagram (Figure 4), it can be further determined that MgAlZn does exist in the alloys with a higher Zn content. Furthermore, the peak intensity of Mg_2_Sn is significantly enhanced with the addition of Zn. This implies that the addition of Zn promotes the precipitation of the Mg_2_Sn phase because of the increase of nucleation sites. 

**Figure 5:**
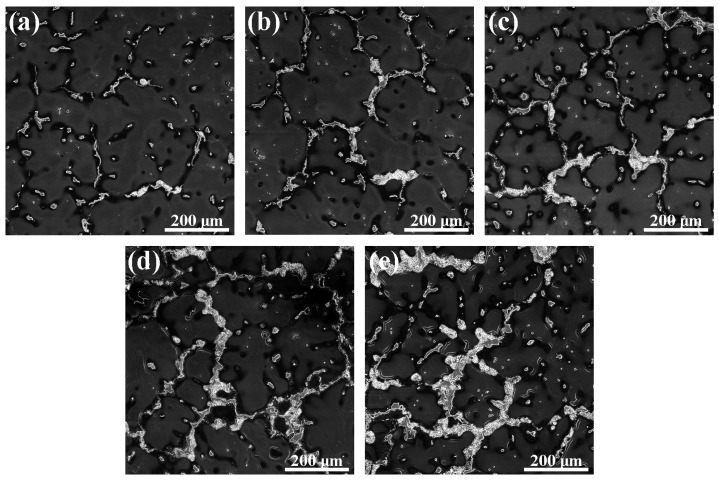
SEM micrographs of the as-cast Mg–6Al–3Sn–0.25Mn–*x*Zn alloys: (**a**) 0 wt %; (**b**) 0.5 wt %; (**c**) 1.0 wt %; (**d**) 1.5 wt %; (**e**) 2.0 wt %.

**Figure 6:**
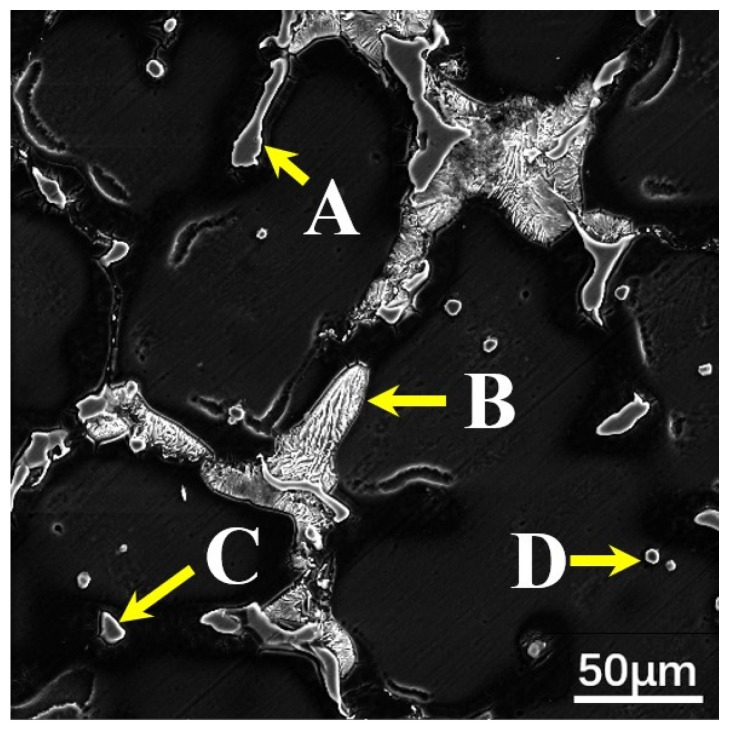
SEM micrographs of the as-cast Mg–6Al–3Sn–0.25Mn–2Zn alloy: (point **A**) MgAlZn phase; (point **B**) a mixture of the MgAlZn phase and Mg_2_Sn phase; (point **C**) MgAlZn phase; (point **D**) Al–Mn phase.

The SEM micrograph and Energy Dispersive Spectrometer (EDS) analysis of the second phase in the Mg–6Al–3Sn–0.25Mn–*x*Zn alloys are shown in Figure 5 and Figure 6, respectively. Four types of second phases are present in the alloys system. For the Mg–6Al–3Sn–0.25Mn alloy, there are mainly Mg_17_Al_12_ phases in the matrix [18]. With the introducing addition of Zn, strip and lamellae structures are observed in the alloys. Combined with XRD results and chemical composition in Table 2, it can be determined that the strip structure is the MgAlZn phase (as shown in point A) [19]. Meanwhile, the lamellae structure is considered to be a mixture of the MgAlZn phase and Mg_2_Sn phase (as shown in point B). Because of the positive enthalpy between Sn with Al and the repulsive interaction between Sn and Zn, the MgAlSn or MgZnSn ternary phases cannot form, but the Mg_2_Sn phase coexists with the Mg_17_Al_12_ and MgAlZn phases [20,21,22]. As the Zn content increases, the strip and lamellae structures gradually develop into coarse network structures, which restrict the growth of the dendrites in the as-cast alloys. It is believed that the coarse eutectic mixture deposits adversely affect the mechanical properties of the as-cast alloys [23]. Additionally, granular second phases are detectable within the grains. Based on the EDS results, the granular second phase is mainly the MgAlZn phase (as shown in point C) and Al–Mn phase (as shown in point D).

**Figure 7:**
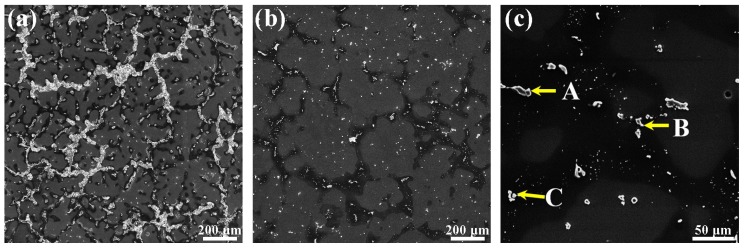
SEM micrographs of Mg–6Al–3Sn–0.25Mn–2Zn alloy: (**a**) as-cast; (**b**) solid-solution state (low magnification); (**c**) solid-solution state (high magnification); (point **A** and point **C**) a mixture of the MgAlZn phase and Mg_2_Sn phase; (point **B**) Al–Mn phase.

In order to confirm the distribution of the second phase after the solution treatment, the microstructure of the Mg–6Al–3Sn–0.25Mn–2Zn alloy is observed, as shown in Figure 7. Compared with the as-cast alloy, the amount of second phases in the solid solution of the Mg–6Al–3Sn–0.25Mn–2Zn alloy are significantly reduced. The bulky strip and lamellae phases are completely dissolved in the matrix, with only a portion of the small particle phase present. The distribution of the second phase becomes more dispersed. It is known from the phase diagram that the Al–Mn phase (as shown in point B) cannot be dissolved into the matrix. The components of these small particles are detected by EDS, and the results are listed in Table 3. In addition to the Al–Mn phase, there are a few MgAlZn and Mg_2_Sn phases (as shown in point A and C) distributed on the grain boundary and inside the grains.

### 3.2. Microstructure Characterization of As-Extruded Mg–6Al–3Sn–0.25Mn–xZn Alloys

**Figure 8:**
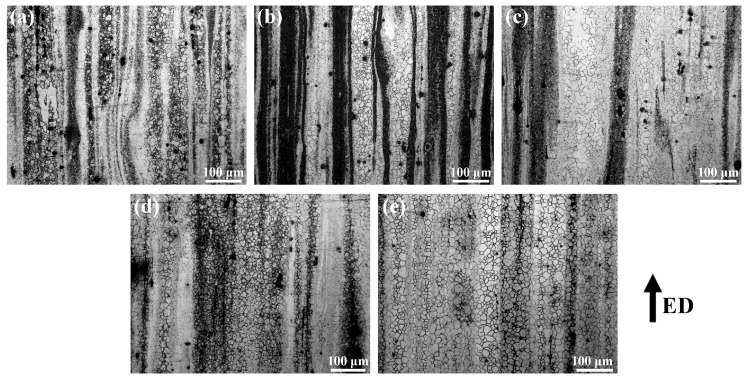
Optical microstructure of the as-extruded Mg–6Al–3Sn–0.25Mn–*x*Zn alloys: (**a**) 0 wt %; (**b**) 0.5 wt %; (**c**) 1.0 wt %; (**d**) 1.5 wt %; (**e**) 2.0 wt %.

Figure 8 displays the optical microstructures of the as-extruded Mg–6Al–3Sn–0.25Mn–*x*Zn alloys in an extrusion–transverse direction (ED–TD) plane. After extrusion, the coarse dendrites of the as-cast alloys are transformed into relatively fine equiaxed structures. For the as-extruded alloys with a low Zn content, there are black band-like regions in the microstructure because of the distribution of the second phase along the ED. In addition, the microstructures of the as-extruded alloys have the characteristics of a bimodal structure, including both recrystallized grains and unrecrystallized grains. With the addition of Zn, the grain size of the as-extruded alloys is slightly coarsened, but the area fraction of the unrecrystallization microstructure is significantly reduced. Among them, the unrecrystallized grains in the Mg–6Al–3Sn–0.25Mn–2Zn alloy completely disappears, and the alloy exhibits a uniform equiaxed grain structure. Most of the second phase tends to be evenly distributed in the Mg matrix. It can be seen that the addition of Zn changes the microstructure of the as-extruded alloys, mainly by affecting the recrystallization mechanism and the distribution of the second phase.

**Figure 9:**
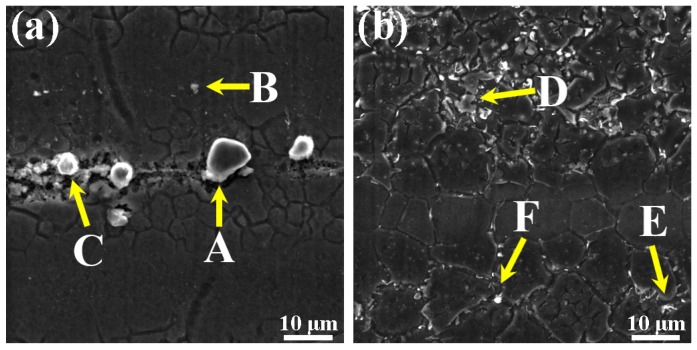
SEM microstructure of the as-extruded Mg–6Al–3Sn–0.25Mn–*x*Zn alloys: (**a**) 0 wt %; (**b**) 2.0 wt %; (point **A** and **C**) Al–Mn phase; (point **B**) Mg_17_Al_12_ phase; (point **D** and **F**) MgAlZn phase; (point **E**) MgAlZn phase which contains the segregation of Sn atoms.

The morphology and distribution of the second phase are significantly changed by adding different Zn contents in the as-extruded alloys (Figure 9). In the Mg–6Al–3Sn–0.25Mn alloy, many large second phases are concentrated along the extrusion direction. These bulky second phases are derived from the Al–Mn phase (as shown in point A and C), which cannot be dissolved into the matrix and breaks up during extrusion. There is also a smaller second phase distributed in the matrix, which is the Mg_17_Al_12_ phase (as shown in point B) containing segregated Sn atoms. With the addition of Zn, the bulk of the second phase substantially disappears, and it is observed that the fine second phase precipitated in the alloys gradually increases and is dispersed in the matrix. This indicates that the addition of Zn promotes the precipitation of the second phase in the as-extruded alloys. From the results of the EDS in Table 4, it can be determined that the MgAlZn phase (as shown in point D and F) mainly exists in the Mg–6Al–3Sn–0.25Mn–2Zn alloy, in which is part of the MgAlZn phase (as shown in point E) and also contains the segregation of Sn atoms. 

## 4. Discussion

### 4.1. Effect of Zn on the Microstructure of the As-Extruded Mg–6Al–3Sn–0.25Mn–xZn Alloys

**Figure 10:**
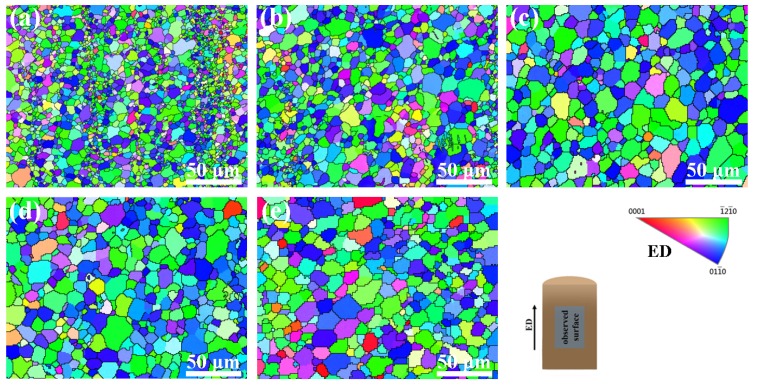
Electron backscatter diffraction (EBSD) inverse pole figure (IPF) maps of the as-extruded Mg–6Al–3Sn–0.25Mn–*x*Zn alloys: (**a**) 0 wt %; (**b**) 0.5 wt %; (**c**) 1.0 wt %; (**d**) 1.5 wt %; (**e**) 2.0 wt %.

In order to further understand the grain distribution of the Mg–6Al–3Sn–0.25Mn–*x*Zn alloys, the EBSD maps taken from the longitudinal section of the as-extruded alloys are presented in Figure 10. For the as-extruded Mg–6Al–3Sn–0.25Mn alloy, there are equiaxed grains in the alloy microstructure, which also contain fine grain bands in the parallel ED. After adding 0.5 wt % Zn, the proportion of fine grain bands is lowered, and the grain size is slightly increased. With the continuous addition of Zn, the fine grain bands finally disappear completely. Although the grains grow up to some extent, the grain sizes are relatively similar. The zinc-free alloy of all of the as-extruded alloys has the smallest grain size, with an average grain size of 3.32 μm. This is related to the highest proportion of fine grain bands in the microstructure. In the Mg–6Al–3Sn–0.25Mn–0.5Zn alloy, the average grain size reaches 4.75 μm, because of the reduced area fraction of the fine grain bands. When the Zn content in the as-extruded alloy is 1.0 wt %, the grain size reaches a maximum with an average size of 9.35 μm. However, as the Zn continued to be added, the grain size of the alloys began to decrease. The average grain size of the Mg–6Al–3Sn–0.25Mn–1.5Zn and Mg–6Al–3Sn–0.25Mn–2.0Zn alloys is 8.98 μm and 8.19 μm, respectively. According to Table 5, the grain size of Mg–6Al–3Sn–0.25Mn alloy has the largest dispersion degree, which is because the alloy has two very distinct grain sizes. In addition, it can be seen that the Zn element changes the grain size by affecting the type and distribution of the second phase in the alloys system. At the same solid solution temperature, the less Zn content in the alloys, the more difficult it is for the bulk Al–Mn phase to be completely dissolved into the matrix. After extrusion, the distribution of the crushed Al–Mn phase is not uniform, and these large-sized second phases not only fail to promote recrystallization nucleation, but also fail to inhibit grain growth. As a result, the grain size near the Al–Mn phase is relatively small, while the coarse grains are in other regions, so the alloys structure forms obvious fine grain bands. With the addition of Zn, not only the bulk Al–Mn phase is solid-dissolved into the matrix, but also the fine and dispersing second phase can be precipitated during the extrusion process. The precipitated second phases serve as the core of the dynamically recrystallized grains nucleation, thereby promoting grain growth. However, when an excessive amount of Zn is added, fine precipitates can also act as pinning grain boundaries to suppress grain growth [24]. This results in a reduced grain size, but it is still larger than alloys with a low Zn content.

### 4.2. Effect of Zn on the Texture of the As-Extruded Mg–6Al–3Sn–0.25Mn–xZn Alloys

**Figure 11:**
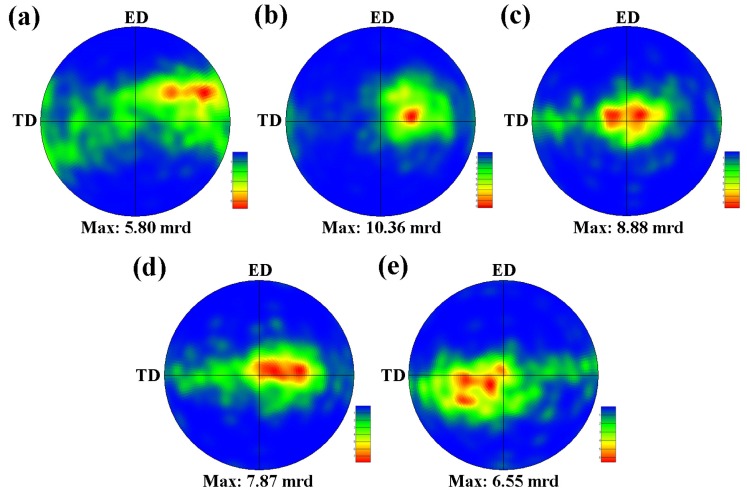
(0001) Pole figures of as-extruded Mg–6Al–3Sn–0.25Mn–*x*Zn alloys: (**a**) 0 wt %; (**b**) 0.5 wt %; (**c**) 1.0 wt %; (**d**) 1.5 wt %; (**e**) 2.0 wt %.

The pole figures of (0001) basal plane are illustrated in Figure 11, revealing the effect of Zn on the texture evolution. The Mg–6Al–3Sn–0.25Mn alloy exhibits a relatively weak texture, for which the basal poles of most of the grains are inclined from the normal direction (ND) to the outside. It is known that the random orientation of the grains is beneficial to start more slip systems and to improve the ductility of the as-extruded alloy. Upon the addition of 0.5 Zn, the texture components begin to become concentrated and the peak intensity increases sharply, indicating that the grains formed a preferential arrangement. With the increase of Zn content, the non-basal texture component gradually disappears, showing that the basal poles of most grains are parallel to the ND. The weakening of the texture peak intensity is attributed to the precipitation of the second phase to promote the recrystallization of the alloys. Eventually, the texture evolves into a typical strong basal texture. In this case, it is difficult to initiate the basal slip by stretching along the ED, as the applied force is parallel to the (0001) basal plane [25,26]. A strong basal texture facilitates the strength of the as-extruded alloys, but loses ductility. In summary, the addition of Zn can change the type of texture and thus affect the mechanical properties of the alloys.

**Figure 12:**
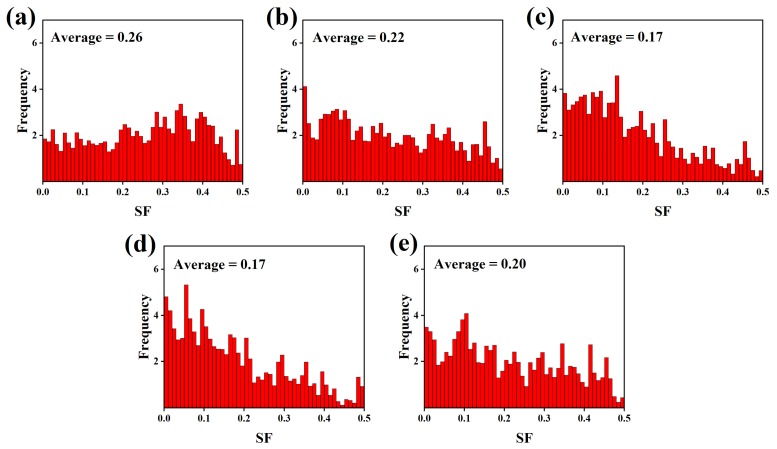
Distribution of the (0001) <1120> basal slip Schmid factor in the as-extruded Mg–6Al–3Sn–0.25Mn–*x*Zn alloys: (**a**) 0 wt %; (**b**) 0.5 wt %; (**c**) 1.0 wt %; (**d**) 1.5 wt %; (**e**) 2.0 wt %.

The Schmid factor is highly dependent on the type of texture. In order to further quantitatively evaluate the effect of the Zn content on the texture of the as-extruded alloys, the Schmid factor of the basal slip is calculated, as shown in Figure 12. The Mg–6Al–3Sn–0.25Mn alloy possesses the largest value of Schmid factors, which means that the orientation of most of the grains belongs to a soft orientation. It is easy to activate the <a> dislocations when stretching along the ED. Therefore, the Mg–6Al–3Sn–0.25Mn alloy will achieve a relatively high ductility, but at the same time, has the lowest yield strength. With the addition of Zn, the grain orientation gradually turns to a hard orientation, leading to an increase in the stress required for starting the basal slip. It is predicted that the yield strength of the alloys improves, but the ductility will deteriorate. Compared with the Mg–6Al–3Sn–0.25Mn–1.5Zn alloy, the Schmid factors value of the Mg–6Al–3Sn–0.25Mn–2.0Zn alloy increases slightly. The main reason is that the excessive precipitation phase can effectively pin the grain boundary and inhibit grain growth. It is not easy to induce twins in the fine grains, which further increases the contribution of the basal slip to plastic deformation.

### 4.3. Effect of Zn on the Recrystallized Fraction of the As-Extruded Mg–6Al–3Sn–0.25Mn–xZn Alloys

**Figure 13:**
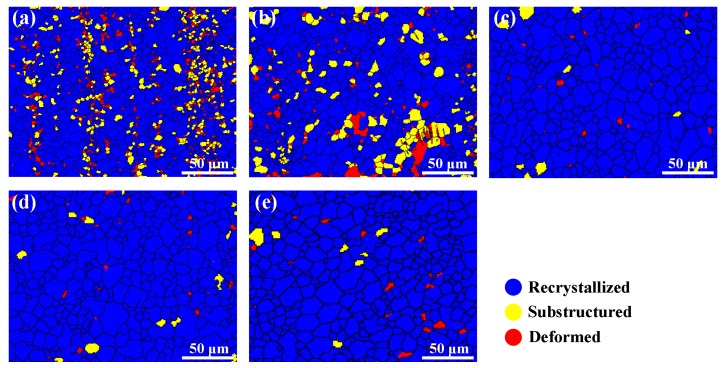
Recrystallized fraction component of the as-extruded Mg–6Al–3Sn–0.25Mn–*x*Zn alloys: (**a**) 0 wt %; (**b**) 0.5 wt %; (**c**) 1.0 wt %; (**d**) 1.5 wt %; (**e**) 2.0 wt %.

The zinc element also affects the complete degree of recrystallization. Figure 13 shows the recrystallized fraction component of the as-extruded Mg–6Al–3Sn–0.25Mn–*x*Zn alloys. The blue areas represent the fully recrystallized grains, while the yellow and red areas represent the sub-grains and deformed grains, respectively. When the Zn content is relatively low, there are a large number of un-recrystallized areas in the microstructure, in which the recrystallization fraction of the Mg–6Al–3Sn–0.25Mn alloy and Mg–6Al–3Sn–0.25Mn–0.5Zn alloy is 82.42% and 82.37%, respectively. With the continuous addition of Zn, the microstructure of the alloy is basically composed of recrystallized components. The difference of the recrystallization fraction is related to the second phase precipitated during the extrusion. Adding the proper amount of Zn to the magnesium alloy can make the precipitated second phase not only small in size, but also evenly distributed in matrix. These fine precipitates have the potential to form nucleation sites and induce recrystallization under the particle stimulated nucleation (PSN) mechanism [27,28,29]. More importantly, the addition of Zn will affect the recrystallization temperature of the Mg–6Al–3Sn–0.25Mn–*x*Zn alloys system. According to the phase diagram, the solidus temperatures decrease gradually with the increase of Zn content, so it can be considered that the addition of Zn promotes the recrystallization process [30,31]. However, the addition of excess Zn has a negative effect on recrystallization, because the large number of precipitates prevent grain boundary migration and growth.

**Figure 14:**
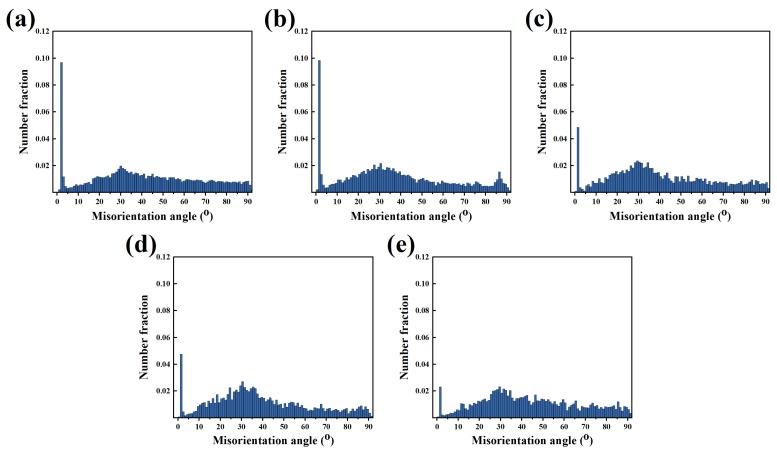
Misorientation angle distributions for the as-extruded Mg–6Al–3Sn–0.25Mn–*x*Zn alloys: (**a**) 0 wt %; (**b**) 0.5 wt %; (**c**) 1.0 wt %; (**d**) 1.5 wt %; (**e**) 2.0 wt %.

To further clarify the recrystallization mechanism of the Mg–6Al–3Sn–0.25Mn–*x*Zn alloys during plastic deformation, the grain boundaries misorientation is calculated as shown in Figure 14. Obviously, the fraction of low angle grain boundaries (LAGBs) exhibits a decreasing trend with the increase of the Zn content. This suggests that Zn promotes the occurrence of continuous dynamic recrystallization (CDRX) during extrusion. It is well known that the dislocation piles up near the second phase to form low angle grain boundaries [32,33,34]. The existing LAGBs continuously absorb dislocations, and thus transform into high angle grain boundaries (HAGBs), eventually forming new recrystallized grains [35]. This indicates that fine precipitation has a positive effect on recrystallization. On the contrary, alloys with a low Zn content have a large number of sub-grains and deformed grains. This is due to the lack of effective recrystallized nucleation sites in the matrix. Therefore, dislocations can only form subgrain boundaries through accumulation and entanglement. 

### 4.4. Effect of Zn on the Tensile Properties of the As-Extruded Mg–6Al–3Sn–0.25Mn–xZn Alloys

**Figure 15:**
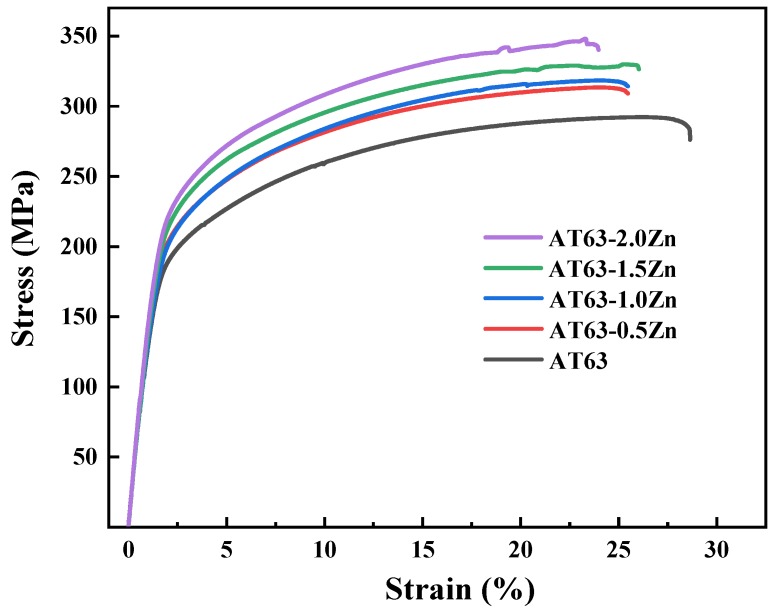
Stress–strain curves of as-extruded Mg–6Al–3Sn–0.25Mn–*x*Zn alloys.

The room temperature tensile stress–strain curves of the as-extruded Mg–6Al–3Sn–0.25Mn–*x*Zn alloys stretched along the ED are given in Figure 15. The corresponding tensile mechanical properties of the alloys are summarized in Table 6, including the yield strength (YS), ultimate tensile strength (UTS), and elongation (EL). It can be seen that the YS and UTS of the alloys increase with the addition of Zn. The Mg–6Al–3Sn–0.25Mn alloy possesses the lowest strength and its YS and UTS are 161 MPa and 292 MPa, respectively. For the Mg–6Al–3Sn–0.25Mn–2.0Zn alloy, the as-extruded alloy exhibits the highest YS and UTS of 206 MPa and 346 MPa. Compared with the alloy without Zn, the YS and UTS are improved by 28% and 19%, respectively. This indicates that Zn has an obvious strengthening effect on the Mg–6Al–3Sn–0.25Mn alloys system. However, the increase in alloy strength is accompanied by a drop in ductility. Among all of the alloys, the Mg–6Al–3Sn–0.25Mn alloy exhibits the best ductility with an EL of 26.7%. After adding Zn, the ductility decreases obviously, and the EL of the Mg–6Al–3Sn–0.25Mn–2.0Zn alloy is only 21.6%, which means that Zn has a negative effect on the ductility of the alloys.

Based on the above discussion, the change of grain size is not the main factor affecting the mechanical properties, because the grain size difference of all of the as-extruded alloys is not significant. The number and distribution of the second phase directly affect the mechanical properties of the alloys system. In the Mg–6Al–3Sn–0.25Mn alloy, the precipitation strengthening has little effect on the improvement of strength, because the number of fine second phases precipitated from the matrix is relatively small. There are some coarse second phases, which are as a result of the breaking of the bulk phase in the as-cast alloys during extrusion. In the tensile process, the coarse second phase causes stress concentration and develops into a crack source, resulting in a low strength of the alloy [36]. In particular, the addition of Zn allows the bulk phase to be solid-dissolved into the matrix, while promoting the precipitation of small-sized precipitates during extrusion. These fine precipitated phases can effectively prevent a dislocation slip so as to achieve precipitation strengthening. It is concluded that the effect of precipitation strengthening with increasing the Zn content is more and more significant. However, the increase in the number of second phases deteriorates the ductility of the alloys to some extent. In addition, the strong basal texture formed in the alloys also contributes to the improvement of strength. In this case, it is difficult to activate the (0001) <1120> basal slip when stretching along the ED, thus the YS is improved.

## 5. Conclusions

In this work, the microstructure and mechanical properties of the Mg–6Al–3Sn–0.25Mn–*x*Zn (*x* = 0, 0.5, 1.0, 1.5, and 2.0 wt %) alloys were systematically investigated. The main conclusions are summarized as follows: 

(1) The as-cast Mg–6Al–3Sn–0.25Mn–*x*Zn alloy exhibits a typical dendritic structure. With the addition of Zn, the dendrite spacing is gradually reduced and the grain size decreases. This is mainly attributed to the addition of Zn to form the MgAlZn phase, while also promoting the precipitation of the Mg_2_Sn phase.

(2) The as-extruded Mg–6Al–3Sn–0.25Mn and Mg–6Al–3Sn–0.25Mn–0.5Zn alloys possess fine grain bands distributed along the ED direction. The Mg–6Al–3Sn–0.25Mn alloy has the finest grains, with an average size of about 3.32 μm. Adding Zn significantly reduces the fine grain bands microstructure and promotes complete recrystallization. The grain size of the Mg–6Al–3Sn–0.25Mn–2.0Zn alloy is about 8.19 μm, and the recrystallization fraction reaches 97.47%. 

(3) Zinc plays an important role in the improvement of strength. When the Zn content is 2.0 wt %, the YS and UTS of the alloy are 206 and 346 MPa, respectively. Compared with the Mg–6Al–3Sn–0.25Mn alloy, the YS and UTS of Mg–6Al–3Sn–0.25Mn–2.0Zn alloy increases by 28% and 19%, respectively. However, the addition of Zn deteriorates the ductility of the alloy to some extent.

## Figures and Tables

**Table 1:** Chemical composition of as-cast alloys.

Nominal Composition	Actual Content (wt %)				
	Al	Sn	Zn	Mn	Mg
Mg–6Al–3Sn–0.25Mn	6.21	2.87	0	0.25	Bal.
Mg–6Al–3Sn–0.25Mn–0.5Zn	6.09	3.08	0.53	0.25	Bal.
Mg–6Al–3Sn–0.25Mn–1.0Zn	6.16	3.22	1.05	0.27	Bal.
Mg–6Al–3Sn–0.25Mn–1.5Zn	6.34	3.31	1.56	0.23	Bal.
Mg–6Al–3Sn–0.25Mn–2.0Zn	6.07	3.22	1.96	0.27	Bal.

**Table 2:** EDS analysis results of second phase in as-cast Mg–6Al–3Sn–0.25Mn–2Zn alloy.

Point	Element (at %)				
	Al	Sn	Zn	Mn	Mg
A	31.70	-	7.36	-	60.94
B	16.37	1.01	2.14	-	90.49
C	32.87	-	6.25	-	60.88
D	48.53	-	-	19.77	31.70

**Table 3:** EDS analysis results of solid-solution state Mg–6Al–3Sn–0.25Mn–2Zn alloy.

Point	Element (at %)				
	Al	Sn	Zn	Mn	Mg
A	27.87	1.14	3.32	-	67.66
B	64.21	1.63	-	22.21	11.94
C	5.35	27.33	-	-	67.32

**Table 4:** EDS analysis results of the as-extruded alloys.

Point	Element (at %)				
	Al	Sn	Zn	Mn	Mg
A	36.09	-	-	21.53	42.38
B	4.67	2.72	-	-	92.60
C	60.25	-	-	36.19	3.57
D	17.20	-	2.84	-	79.96
E	15.70	1.80	2.33	-	80.17
F	23.77	-	3.23	-	72.99

**Table 5:** Grain size of as-extruded Mg–6Al–3Sn–0.25Mn–*x*Zn alloys.

Alloy	Average Grain Size (μm)	*σ*	Coefficient of Variation (*σ*/Average Grain Size)
Mg–6Al–3Sn–0.25Mn	3.32	2.82	0.85
Mg–6Al–3Sn–0.25Mn–0.5Zn	4.75	3.94	0.83
Mg–6Al–3Sn–0.25Mn–1.0Zn	9.35	6.55	0.70
Mg–6Al–3Sn–0.25Mn–1.5Zn	8.98	6.56	0.73
Mg–6Al–3Sn–0.25Mn–2.0Zn	4.83	8.19	0.59

**Table 6:** Tensile mechanical properties of the as-extruded Mg–6Al–3Sn–0.25Mn–*x*Zn alloys at room temperature.

Alloy	YS (MPa)	UTS (MPa)	EL (%)
Mg–6Al–3Sn–0.25Mn	161 ± 4	292 ± 3	26.7 ± 1.4
Mg–6Al–3Sn–0.25Mn–0.5Zn	173 ± 2	313 ± 2	23.1 ± 0.5
Mg–6Al–3Sn–0.25Mn–1.0Zn	177 ± 3	317 ± 3	23.2 ± 0.9
Mg–6Al–3Sn–0.25Mn–1.5Zn	194 ± 4	329 ± 1	23.4 ± 1.0
Mg–6Al–3Sn–0.25Mn–2.0Zn	206 ± 4	346 ± 4	21.6 ± 0.7
